# The Impact of Metacognitive Beliefs on Sexual Functions and Satisfaction in Vaginismus

**DOI:** 10.1192/j.eurpsy.2025.2318

**Published:** 2025-08-26

**Authors:** S. Önen, M. Aydın, G. Özdamar Ünal

**Affiliations:** 1 Department of Psychiatry, University of Health Sciences, Bursa Faculty of Medicine, Bursa; 2 Department of Psychiatry, Selcuk University Faculty of Medicine, Konya; 3 Department of Psychiatry, Süleyman Demirel University Faculty of Medicine, Isparta, Türkiye

## Abstract

**Introduction:**

Vaginismus is characterized by phobic avoidance, involuntary pelvic muscle contraction, anticipation, fear, and experience of pain during vaginal penetration. In addition to anxiety and fear, vaginismus-specific cognitive and metacognitive beliefs are thought to play a role in the etiology of vaginismus. Impaired sexual functions and decreased sexual satisfaction in women with vaginismus are claimed to be associated with anxiety and depressive symptoms. However, in clinical practice, it is observed that women who do not exhibit anxiety and depressive symptoms also experience sexual dysfunction and reduced sexual satisfaction, but it is noteworthy that the causes of this deterioration have not been sufficiently investigated.

**Objectives:**

The purpose of this study is to assess the impact of metacognitive beliefs on sexual functions and satisfaction in women with vaginismus.

**Methods:**

A total of 64 women with vaginismus and 30 healthy controls were examined through Sociodemographic Data Form (including age, education status, duration of marriage, etc), Beck Depression Inventory (BDI), Beck Anxiety Inventory (BAI), Arizona Sexual Experiences Scale (ASEX), Golombok-Rust Inventory of Sexual Satisfaction (GRISS), and Metacognition Questionnaire-30 (MCQ-30).

**Results:**

The mean ASEX, GRISS, and MCQ-30 scores were significantly higher in the vaginismus group than the healthy controls. No significant difference were found between groups in terms of BDI and BAI scores. Hierarchical Regression Analysis revealed that 13% of ASEX total scores in the vaginismus group were predicted by BDI and BAI scores (F=4.59, p< 0.05), and the predictability increased significantly to 32% by the addition of MCQ-30 scores to the model (F=3.79, p<0.01). However, GRISS-Total scores were not statistically significantly predicted by BDI and BAI scores (F=1.76, p>0.05), but the predictability of variance increased significantly to %26 (F=2.87, p<0.05) with the addition of MCQ-30 scores to the model. Moreover, the metacognitive dimension of uncontrollability and danger of thoughts, and cognitive self-consciousness were found to be significant factors in predicting both ASEX (*b*=0.52, p=0.004 and *b* =-0.49, p=0.003, respectively) and GRISS (*b*=0.58, p=0.002 and *b* =-0.40, p=0.017, respectively) scores in vaginismus.

**Conclusions:**

The current findings of the study indicate that metacognitive beliefs, especially dimensions of uncontrollability and danger of thoughts and cognitive self-consciousness, predict sexual functioning and sexual satisfaction in women with vaginismus. Understanding the metacognitive characteristics accompanying vaginismus and including metacognitive interventions in sexual therapy for both cognitive self-consciousness and negative beliefs about the uncontrollability of thoughts and danger, may result in increased treatment success, improved sexual functioning, and sexual satisfaction in women with vaginismus.

**Disclosure of Interest:**

None Declared

